# Localization of the ilioischial line on axial computed tomography images for preoperative planning of total hip arthroplasty

**DOI:** 10.1186/s12891-022-06021-1

**Published:** 2022-11-30

**Authors:** Michitaka Kato, Hideki Warashina, Akito Kataoka, Takanori Ando, Shingo Mitamura

**Affiliations:** Nagoya Joint Replacement Orthopaedic Clinic, Iponbashi, Takadaji, Kita-Nagoya, Aichi 481-0011 Japan

**Keywords:** Ilioischial line, Pelvic radiograph, Axial computed tomography, Total hip arthroplasty, Cup offset

## Abstract

**Background:**

If the bony region indicating the ilioischial line is established on the preoperative axial computed tomography (CT) image, the distance between the simulated cup and the ilioischial line can be measured on this image so that the surgeon can use these data to define a more accurate preoperative two-dimensional (2D) template of total hip arthroplasty (THA). This study aimed to verify the hypothesis that on the CT axial image, the cortical bone area, indicated by the superimposition of a line (line α) with a perspective projection angle to the ilioischial line on radiography and tangent to the medial acetabular wall, is the cortical bone that represents the ilioischial line on radiography.

**Methods:**

Study 1: If the two measured distances (distance A’ and distance B) are sufficiently equal, then the hypothesis can be supported. Distance A’ was calculated by multiplying the distance A, between the ilioischial line and the medial margin of the metal cup after THA measured at the level of the hip joint center on the pelvic radiograph, by 0.91 to correct for radiographic magnification. Distance B was defined as the distance between the medial margin of the metal cup and line α on the axial CT image at the level of the hip joint center. These two distances were measured for all 51 hip joints included in the study.

Study 2: The difference between distance A and distance A^#^ (distance A on the 2D template) was compared between the group containing 59 primary THAs in which distance B′ was measured (distance B in the simulation) and the control group containing 59 primary THAs.

**Results:**

Study 1: The average distance for A’ was 4.5 ± 2 mm, and the average distance for B was 4.7 ± 2.1 mm. The difference between distances A and B was 0.2 ± 0.2 mm.

Study 2: The mean difference between distance A and distance A^#^ for the measurement and control groups was 1.8 ± 1.3 mm and 3.7 ± 2.4 mm, respectively (*P* < 0.001).

**Conclusions:**

The ilioischial line is located in the bony region where line α intersects the medial acetabular wall with a maximum overlap on axial CT images.

**Supplementary Information:**

The online version contains supplementary material available at 10.1186/s12891-022-06021-1.

## Background

The ilioischial line is very conspicuous on pelvic radiographs and is often used as an anatomical landmark. It is also a reference line for characteristic hip morphology in coxa profunda [1–3] and protrusio acetabuli [4–6]. Preoperative planning of total hip arthroplasty (THA) with pelvic radiographs is a standard procedure. In this regard, the ilioischial line has been suggested as a reference for cup placement [7], likely because it is recognized as a representative part of the medial acetabular wall [8, 9]. However, to date, no report has described in detail the location of the bony region indicated by the ilioischial line on axial computed tomography (CT) images.

**Fig. 1:**
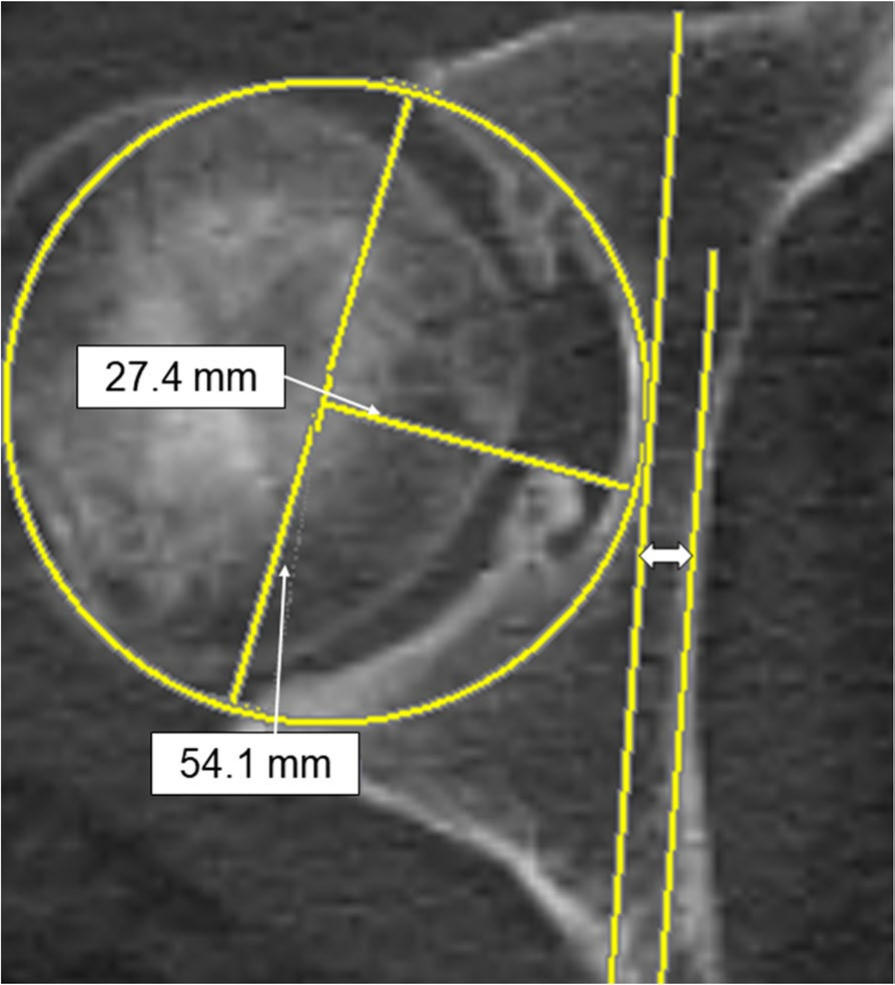
Preoperative planning based on CT images. The slice of the femoral head with the largest AP diameter on CT axial images is selected as the hip center, with the cup diameter estimated by using the distance between the edge of the anterior wall and the edge of the posterior wall of the acetabulum. The depth where the cup is placed is predicted from the radius of the acetabulum. If the position of the ilioischial line on the CT image is known, information concerning the offset of the cup could be obtained. “⇔” indicates the expected distance from the ilioischial line to the cup. CT, computed tomography

**Fig. 2:**
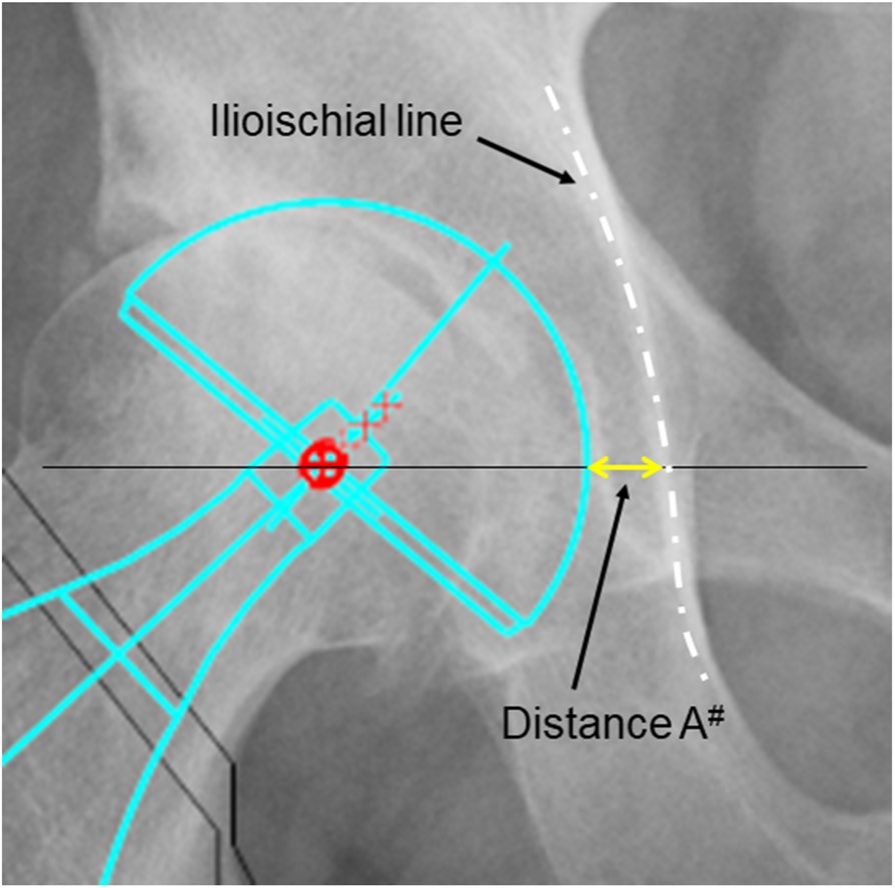
Two-dimensional templating. The image shows the usage of the ilioischial line for determining the target offset position of the acetabular cup. The exact distance from the ilioischial line to the acetabular cup is unclear on CT axial images. CT, computed tomography

Previous studies have created a two-dimensional (2D) template on radiographs before THA, in which preoperative pelvic CT images are used to confirm the cup size and the combined anteversion [10, 11]. The cup size is determined by the anteroposterior (AP) width of the acetabulum and measured at the level of the femoral head center within the anatomical hip center on CT images (Fig. 1). When a cup with an appropriate size is placed on the anterior and posterior walls on the axial CT in the preoperative simulation, the distance between the medial margin of the cup and the ilioischial line can be measured if the bony area showing the ilioischial line on the CT images is known (Fig. 1). The distance (distance A^’^) from the ilioischial line to the cup can be reflected as the offset position of the cup in the 2D template, as shown in Fig. 2.

In this study, we identified the bony region of the ilioischial line on axial CT using postoperative pelvic radiographs and CT images. On the pelvic radiographs, the distance from the metal cup to the ilioischial line was measured in a supine pelvic radiograph considering its magnification rate. If this actual distance and the distance between the medial margin of the metal cup and the medial acetabular wall, which shows the hypothetical ilioischial line on axial CT, were equal, the bony region of the medial acetabular wall would be equivalent to the ilioischial line on the CT image.

The study aimed to validate the hypothesis that the bony region on CT axial images, which overlaps with the line (line α) with a perspective projection angle to the ilioischial line during radiography and with a tangent to the medial acetabular wall, represents the ilioischial line on X-ray. Further, this study attempted to verify the accuracy of the cup offset distance of the preoperative 2D template created using this information.

## Methods

### Study 1

#### Study design

This retrospective evaluation using plain radiographs and axial CT findings of patients was approved by the Institutional Review Board of the Nagoya Orthopedic Joint Replacement Clinic (Approval No. 201905002). Written informed consent was obtained from all patients for THA and for the publication of THA results.

#### Location of the ilioischial line

**Fig. 3:**
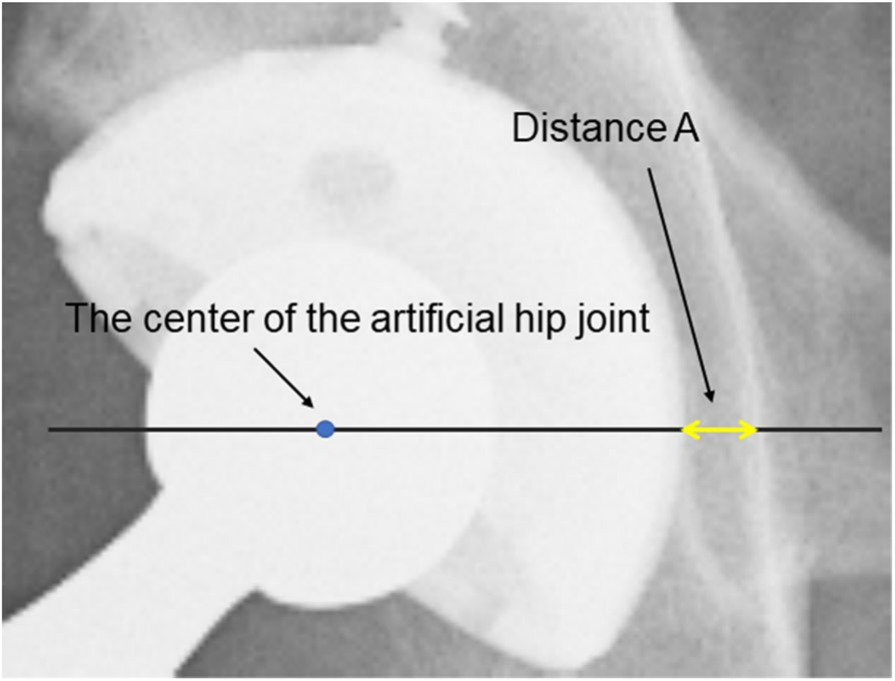
The distance from the medial margin of the acetabular cup to the ilioischial line measured along a line parallel to the inter-teardrop line and through the center of the femoral head

**Fig. 4:**
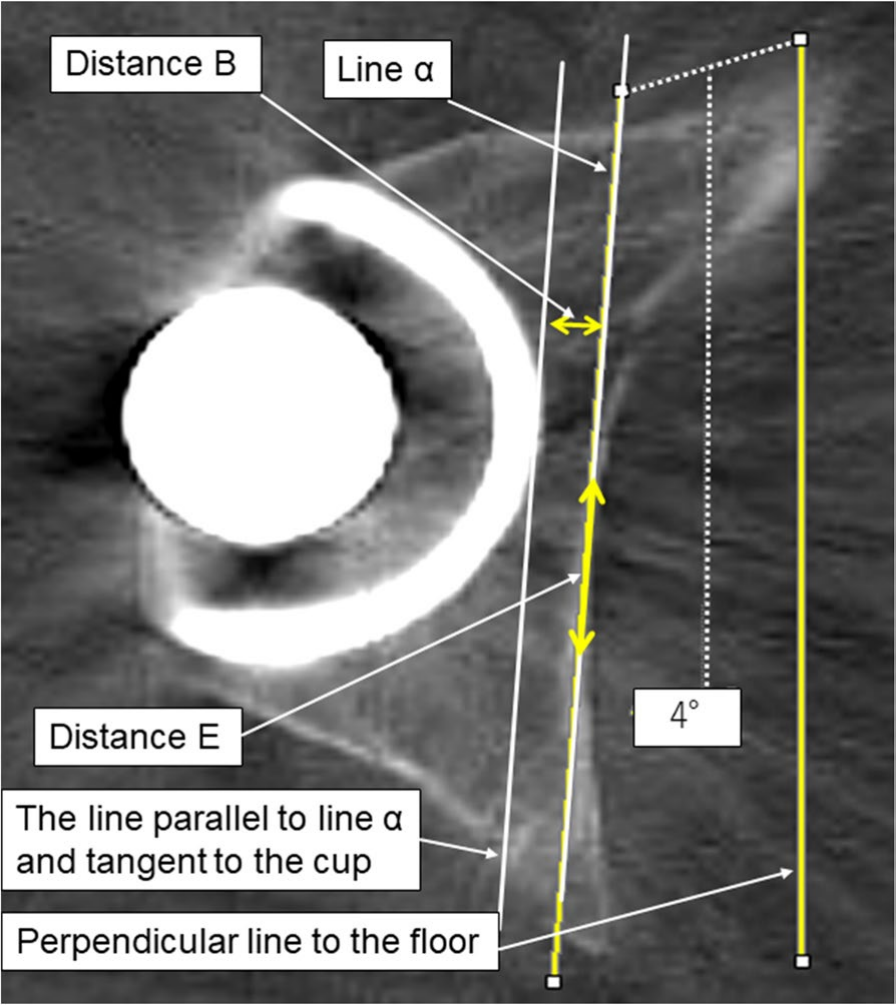
Measurement of distance B. In the axial CT image at the level of the hip joint center, the distance between the metal cup and line α tangent to the medial acetabular wall with maximum overlap was measured as shown. CT, computed tomography

During radiography, the angle between the X-ray beam from the X-ray tube to the ilioischial line and a line perpendicular to the floor was calculated using a trigonometric function. We assumed that the bony region on the CT axial image that most overlapped with line α defines the bony region of the ilioischial line. This hypothesis would be proven if the following two distances were equivalent when measured with the patient in the supine position: Distance A’ calculated by multiplying distance A (Fig. 3) between the ilioischial line and the medial margin of the metal cup measured at the level of the hip joint center on the pelvic radiograph by 0.91 to correct for radiographic magnification, and Distance B measured between line α and the medial margin of the metal cup on axial CT images (Fig. 4).

#### Study sample

The study enrolled 67 patients (76 hip joints) who underwent primary THA performed by one of the authors between November 2018 and September 2019. The exclusion criteria were as follows: absence of postoperative CT images or supine radiographs of the pelvis; Crowe classification III and IV; radiographs in which the distance between the cup and ilioischial line could not be measured because of contact or overlap; cementless cup fixation in which screw fixation interfered with measurements; use of a cemented cup; pelvic osteotomy; and pelvic fracture. After applying the exclusion criteria, 44 patients (51 hip joints) were included (12 male patients), with a mean age (±standard deviation) of 63.2 ± 8.4 years and a body mass index (BMI) of 24.6 ± 4 kg/m^2^.

#### Imaging

Plain pelvic radiographs and CT axial images were obtained routinely on postoperative day 5. Radiographs were taken with the patient in the supine position. The X-ray tube was positioned vertically to the floor and centered on the pubic symphysis, with a film focal length of 100 cm. Three observers measured the distance from the center of the femoral heads to the floor on the CT axial images. Imaging results from 44 cases showed good reliability (inter-class correlation coefficient [ICC], 0.85). The average distance from the hip joint to the film was 10.7 cm, corresponding to a magnification of approximately 110% on the radiographs.

Routine CT examinations were performed to evaluate occult fractures and the cup and stem anteversion position. A 16-row multi-CT model ECLOS (HITACHI, Tokyo) was used for all patients. Multiplanar Reformation axial images at 3-mm intervals from the volumetric CT were created parallel to the inter-teardrop line. The slice with the largest AP diameter of the metal femoral head was selected to determine the location of the ilioischial line.

#### Angle of the line of radiation

**Fig. 5:**
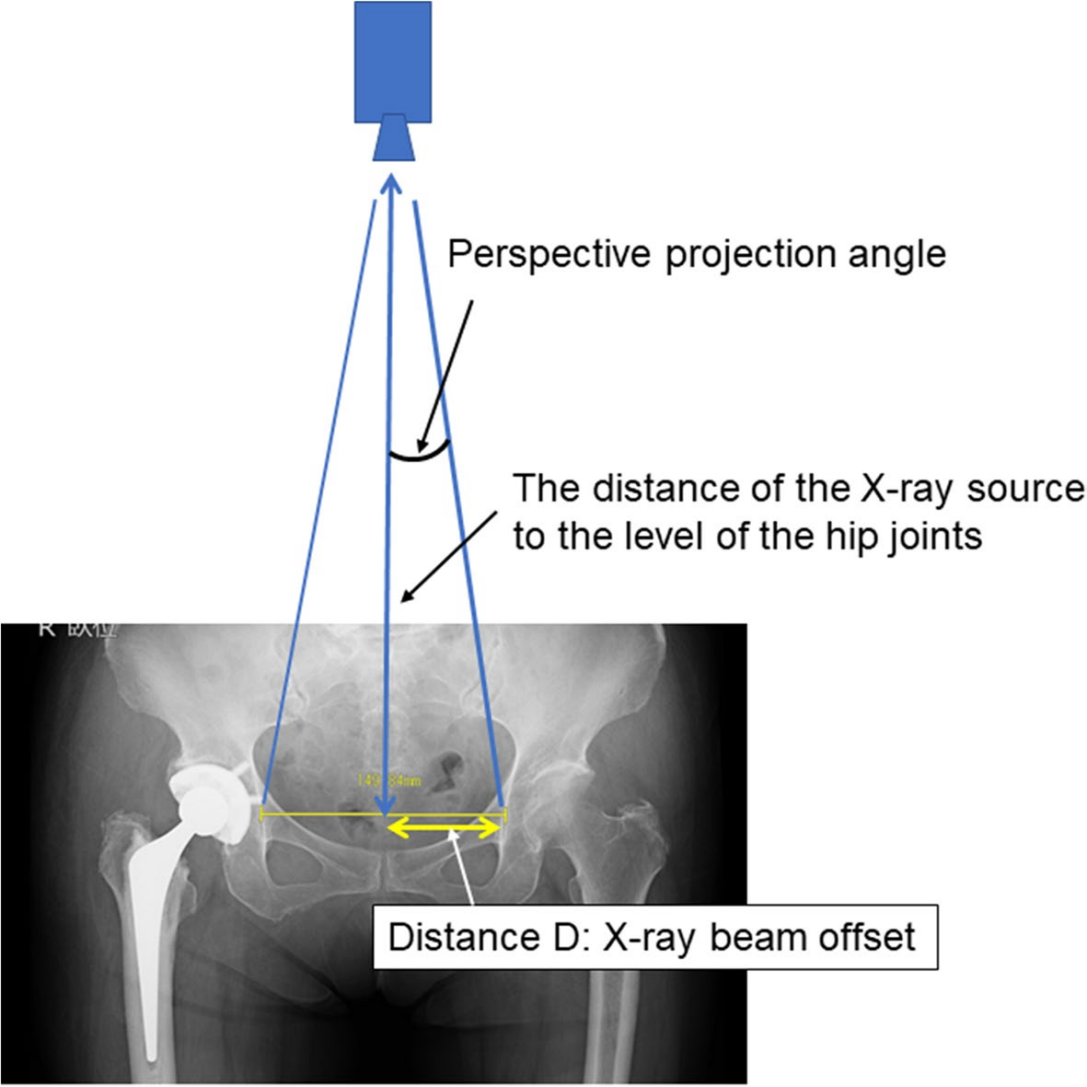
The offset of the X-ray beam measured on an AP radiograph of both hips. The X-ray beam focused on the pubic symphysis. In this figure, the X-ray beam offset angle in the ilioischial line can be calculated by a trigonometric function, using the distance from the center of the pelvis to the ilioischial line and the distance from the X-ray source to the film. With this method, an X-ray beam offset angle of approximately 4° is located around the ilioischial line

The perspective projection angle of the X-ray beam around the ilioischial line was calculated as follows (Fig. 5): tan θ = − 1× (distance C from the radiation source to the film/distance D between the ilioischial lines/2). Distance C was set to 100 cm. Distance D, positioned between the right and left ilioischial lines, was measured twice by four observers on the AP radiographs for each hip as a reference. The inter- and intra-observer reliabilities were excellent (ICC, 0.99). The mean distance between the two ilioischial lines was 133.5 mm, with half the distance (6.7 cm) used as distance D to calculate tan θ. The perspective projection angle of the X-ray beam to the ilioischial line was calculated to be approximately 3.8°. Therefore, the angle was set to 4° to the vertical line for simplicity.

#### Measurement of distance a, distance B, and distance E

Distance A from the ilioischial line to the margin of the metal cup, at a level through the femoral head center and parallel to the inter-teardrop line on the pelvic radiograph in the supine position, was measured to one decimal place in mm using a digital viewer (Fig. 3). The viewer image was enlarged sufficiently for this measurement. Since the radiographic magnification was approximately 110%, the measured distance A was multiplied by 0.91 to obtain distance A’, which was the actual distance.

When measuring distance B, we selected the slice that showed the largest metal femoral head because that slice was considered to be the closest to the center of the femoral head. A line perpendicular to the floor was drawn. Subsequently, another line with a 4° inclination to the perpendicular line was drawn. If the line were not perpendicular, it would appear jagged with intermittent steps on the digital viewer. Therefore, by drawing a straight line without any steps, it would be perpendicular to the floor. This line with a 4° inclination was the line that would be projected radiographically from the X-ray tube to the hip joint. This line was slid to the position tangent to where the medial acetabular wall overlapped the most and was defined as line α. Distance B was measured between line α and another line that was parallel to line α and tangent to the medial margin of the metal cup (Fig. 4). Distance E, the distance of the cortical bone area, in which line α overlapped the medial acetabular wall, was also measured on CT axial images (Fig. 4).

**Fig. 6:**
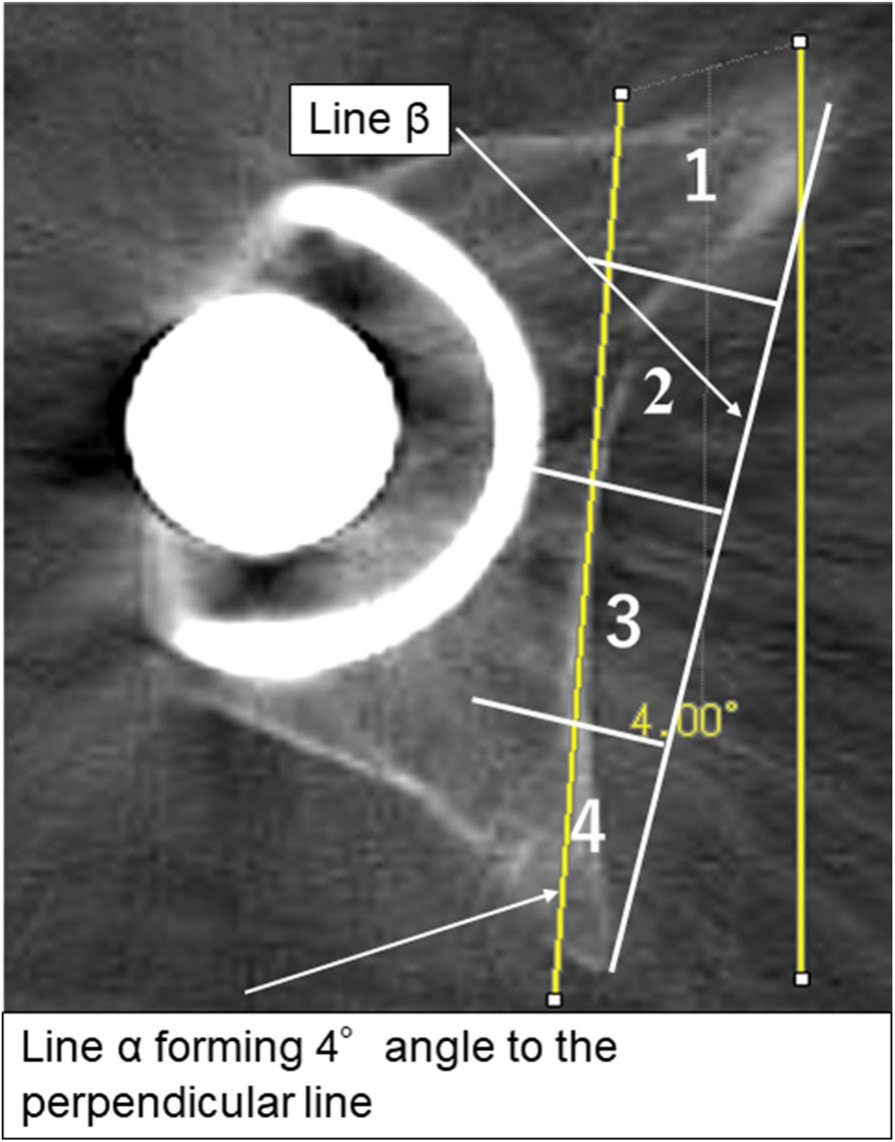
Location of the ilioischial line on the medial acetabular wall. The line connecting the anterior and posterior margins of the medial acetabular wall is divided into four equal parts (i.e., zones 1–4) from the anterior to the posterior point. A perpendicular line is drawn through all the zones and the medial acetabular wall. In this example, the center of the medial acetabular wall that overlaps with line α is located in zone 3

To determine whether the ilioischial line was located anteriorly or posteriorly within the medial acetabular wall, we drew line β from the most anterior to the most posterior point on the medial acetabular wall. Then, this line was divided into four equal parts, designated as zones 1–4, from the anterior to the posterior point (Fig. 6). The observer investigated in which zone the center of the cortical bone region where line α overlaps the medial acetabular wall is located. If the center of the cortical bone was located between two zones, the two adjacent zones were identified.

#### Statistical analysis

For the 51 hips, distances A, B, D, and E and zone classification of the location of the ilioischial line were recorded in duplicates by four blinded observers (one orthopedic surgeon, two physical therapists, and one radiological technician), each with at least 15 years of clinical experience, with an interval of at least 2 weeks between measurements. Images were fully magnified with a digital viewer and measured to one decimal place in mm. Statistical analyses were performed using SPSS® software (version 26.0; IBM Corp., Armonk, NY, USA). The ICC was calculated for intra- and inter-observer reliability. ICC values were interpreted as follows [12]: < 0.5, poor reliability; 0.5–0.75, moderate reliability; 0.75–0.9, good reliability; and > 0.90, excellent reliability. Data were expressed as means (±standard deviations [SDs]), with a total of 408 measures (four observers, measured twice, 51 hips). The difference between distances A’ and B was calculated for each hip. The percentage of hips in which this difference was > 1 and > 2 mm was calculated. The correlation coefficient between distances A’ and B was calculated. A Bland–Altman analysis was used to determine the level of agreement between the measured distances. The distribution of localization of the ilioischial line within the four zones of the medial acetabular wall was calculated.

### Study 2

We retrospectively investigated whether this method improved the accuracy of cup offset when creating a preoperative template. The group that underwent primary THA after creating a 2D digital template using Advanced Case Plan® (Stryker Imaging, Flower Mound, TX, USA) by measuring the size of the cementless cup from the anteroposterior diameter of the acetabulum on a preoperative CT axial image at the original hip center was defined as the control group. The cup size and distance B were measured based on the preoperative CT axial images, and the distance A^#^ was obtained by multiplying distance B with a magnification factor of 1.1 during radiography. The group that underwent primary THA after creating a 2D digital template using the distance A^#^ and cup size data was defined as the measurement group. Cases with missing data and Crowe types 2, 3, and 4 were excluded. The differences between distance A^#^ on preoperative 2D digital templates stored on a PACS (Konica Minolta, Tokyo, Japan) server and distance A in the postoperative pelvic AP radiographs were compared.

This study included 59 patients who underwent consecutive primary THA from April 2020 to March 2021 (sex, 11 men and 48 women; mean age, 63.1 ± 9.1 years; mean BMI, 25.2 ± 4.8 kg/m^2^) in the measured group. Moreover, another 59 patients who underwent consecutive primary THA from February 2018 to December 2018 were included in the control group (sex, 12 men and 47 women; mean age, 65 ± 9.4 years; mean BMI, 23.9 ± 4.5 kg/m^2^). An independent t-test was used to determine the significance level, which was set at < 5%. The effect size was calculated to be 0.88 using the G*power software (version 3.1.9.7; Universitat Dusseldorf, Dusseldorf, Germany) from the means and standard deviations of the two groups. The power was calculated to be 0.997 based on the effect size and the number of samples, indicating that the sample size was sufficient.

## Results

### Study 1

The inter- and intra-observer reliabilities in measurement were excellent for distances A, B, and D. However, for distance E, although the intra-observer reliability was good, the inter-observer reliability was only fair for the first measurement and moderate for the second (Table 1).

**Table 1:** Inter- and intra-observer agreements (ICC [2, 1] and ICC [1], respectively) for the measurement of the A, B, D, and E distances

		First measurement	Second measurement		Observer 1	Observer 2	Observer 3	Observer 4
Distance A	ICC (2,1)	0.96	0.99	ICC (1,1)	0.98	0.93	0.99	0.98
	Lower bound	0.94	0.98	Lower bound	0.97	0.88	0.98	0.97
	Upper bound	0.98	0.99	Upper bound	0.99	0.96	0.99	0.98
Distance B	ICC (2,1)	0.96	0.96	ICC (1,1)	0.98	0.96	0.99	0.98
	Lower bound	0.93	0.92	Lower bound	0.96	0.93	0.97	0.97
	Upper bound	0.97	0.98	Upper bound	0.99	0.98	0.99	0.99
Distance D	ICC (2,1)	0.98	0.91	ICC (1,1)	0.99	0.97	1	0.84
	Lower bound	0.96	0.87	Lower bound	0.99	0.95	0.99	0.73
	Upper bound	0.99	0.94	Upper bound	1	0.98	1	0.90
Distance E	ICC (2,1)	0.44	0.57	ICC (1,1)	0.72	0.80	0.77	0.74
	Lower bound	0.21	0.34	Lower bound	0.56	0.68	0.63	0.59
	Upper bound	0.64	0.73	Upper bound	0.83	0.88	0.86	0.84

ICC, intraclass correlation coefficient

**Fig. 7:**
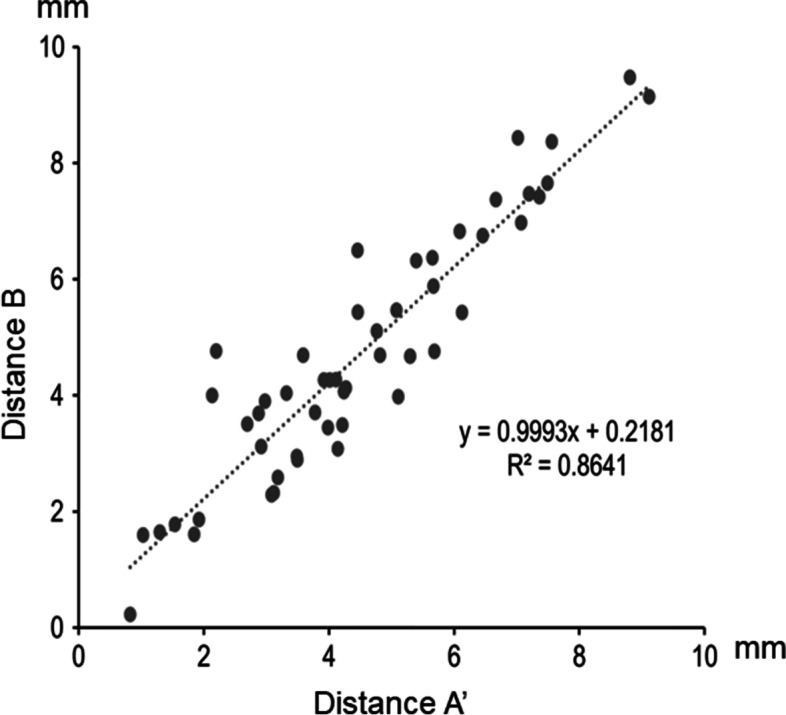
Scatter plot of measured distances A’ and B for each joint. The correlation between distances A’ and B is high (R^2^ = 0.86). The regression line is shown as (y = x + 0.2)

**Fig. 8:**
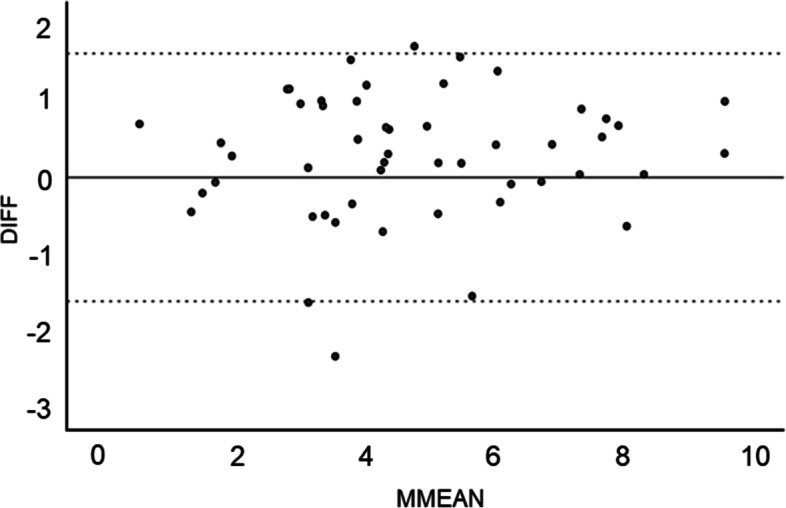
Bland–Altman plot of the measurement error. The measurement error exceeds the allowable range in only three cases

The average distances A’ and B for the 51 joints were 4.5 ± 2 and 4.7 ± 2.1 mm, respectively. The mean difference between distances A’ and B was 0.2 ± 0.2 (range, 0–2.6) mm (see Additional file 1). The scatter plot of distance A’ vs. distance B for each case is presented in Fig. 7. The correlation between distance A’ and distance B was strong (R^2^ = 0.86). A difference of ≥1 mm between the two distances was observed in 11 cases (21.6%). A difference of ≥2 mm was observed in one case (2%). The Bland–Altman plot shows that 94.1% of the differences in measurements were within ±1.96 SD, with only three outliers (Fig. 8).

**Fig. 9:**
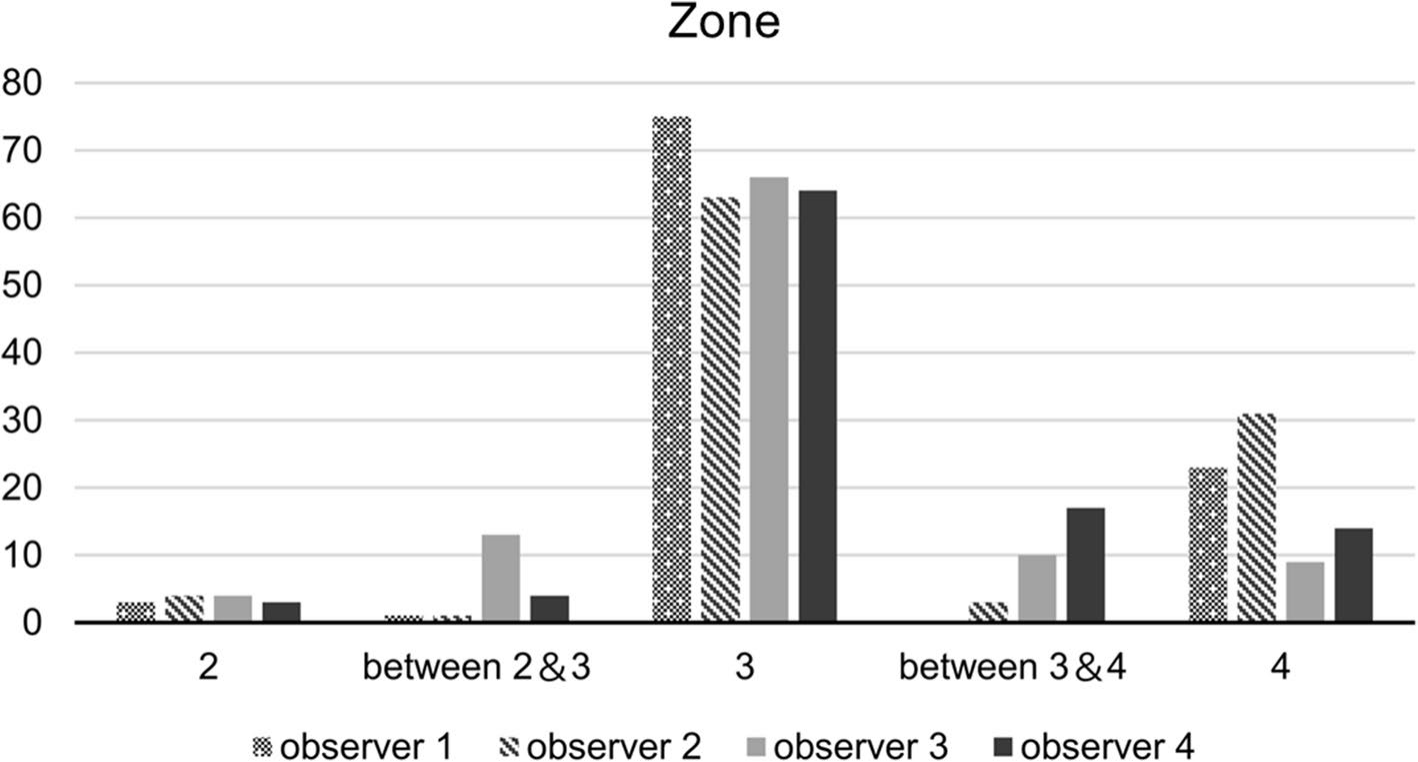
Location of the cortical bone of the medial acetabular wall. The ilioischial line is located in zone 3 of the medial acetabular wall in 65.7% of cases and posterior to the medial acetabular wall in 91.9% of cases

The mean distance E for the 51 joints was 22.1 ± 5.1 (range, 13.7–35.8) mm (see Additional file 1). The distribution of the 408 localizations of the ilioischial line within the four zones of the medial wall of the acetabulum was as follows (Fig. 9) (see Additional file 1): 0 (0%) in zone 1; 14 (3.4%) in zone 2; 19 (4.7%) between zones 2 and 3; 268 (65.7%) in zone 3; 30 (7.4%) between zones 3 and 4; and 77 (18.4%) in zone 4. Overall, the ilioischial line was located in the posterior half of the medial acetabular wall in 92% of cases (Fig. 9).

### Study 2

The mean difference between distance A^#^ and distance A was 1.8 ± 1.3 mm in the measured group and 3.7 ± 2.4 mm in the control group (see Additional file 1). The difference between distance A^#^ and distance A was significantly smaller in the measured group than in the control group (*p* < 0.001).

## Discussion

In recent years, three-dimensional templating has been used for preoperative planning of THA procedures [13, 14]. However, this method requires specialized software and is time-consuming. Conversely, 2D preoperative templating using plain radiographs and axial CT images is simple, inexpensive, and can be completed within a few minutes. Knowing the ilioischial line on CT images can provide information on the offset for cup placement, which may help creat more accurate 2D radiographic templates. This hypothesis was proven in Study 2. To avoid iliopsoas impingement [15–19] and ensure adequate acetabular coverage [7, 20] and THA stability [21–23], the offset position of the cementless cup is critical. Upon placement, the anterior edge of the cup should not hang over the anterior acetabular wall. Additionally, the host bone above the cup should be adequately covered to prevent the cup from loosening. For patients with acetabular dysplasia, where the center-to-edge angle of the cup is < 0°, the surgeon should consider placing the cementless cup more medially or with the hip center positioned slightly higher to stabilize the cup [23]. A cemented cup could be used to graft the bulk bone of the acetabulum if necessary [24, 25]. The cup size can be predicted at the level of the acetabulum using CT. The distance from the ilioischial line on CT to the cup can be measured and incorporated into the radiographic template to adjust for the simulation, particularly in patients with acetabular dysplasia. Global offset is also vital for hip function [26]. However, the need for cup medialization may require the selection of a high offset stem.

The ilioischial line is also an important landmark for identifying hip joint morphologies, including coxa profunda and protrusio acetabuli. In their CT-based imaging study, O’Sullivan et al. [8] identified the location of the ilioischial line in 10 cadaveric pelvises to be posterior to the acetabulum and at the radiographic interface of the cortical bone of the posterior column. However, a 5° rotation of the pelvis yielded a change in the radiographic location of the teardrop used as a reference for the ilioischial line. In their investigation of the morphology of the acetabulum in coxa profunda, Fujii et al. [9] reported that the ilioischial line was located at the outermost point of the sciatic acetabular medial wall. We hypothesized that the bony region on CT axial image, which overlaps line α, presented a perspective projection angle to the ilioischial line running tangent to the medial acetabular wall. This region was the bony region of the ilioischial line on X-ray. This approach required a consideration of the perspective projection angle to the ilioischial line. Similarly, the X-ray beam offset has previously been used to measure acetabular cup anteversion after THA [26–30]. Goergen and Resnick proposed a correction factor of 5° for the radiographic angle for AP pelvis radiographs centered on the symphysis pubis. However, this correction factor was variable for different relative cup positions [29]. Using trigonometry, Widmer calculated a radiographic beam offset of 5.46° for measuring the cup anteversion angle [27]. By measuring the distance from the midline to the ilioischial line on AP radiographs from patients in the supine position, we calculated a radiographic angle of 4°. While our hypothesis of the relationship between line α and the ilioischial line is theoretical, it is consistent with previous opinions [8, 9]. By confirming that distances A’ and B were nearly equivalent, our study aimed to demonstrate that line α is the bony region of the ilioischial line on axial CT images. We showed that these two distances were strongly correlated (R^2^ = 0.87) and had an average difference of 0.2 mm (calculated from the regression line y = x + 0.2). The ilioischial line was located in the cortical bone area of the medial acetabular wall that overlapped the most with line α. The average width of the cortical bone defining the ilioischial line was approximately 2 cm. However, the measurement reliability was only fair-to-moderate. The zone classification showed that the ilioischial line was concentrated in zone 3 and was located posteriorly in almost 90% of cases. A more posterior zone containing the ilioischial line indicates a shorter cup distance. Nonetheless, we have excluded cases where the cup and ilioischial line either touched or overlapped. Therefore, there was a possibility for localization of the ilioischial line in zone 4 in some cases.

Measurement errors of distances A and B can result from the magnification rate of the X-ray beam, the differences in pelvic rotation in the supine position during imaging, and the metal artifacts in the cup. O’Sullivan et al. [8] reported that the relationship between the ilioischial line and teardrop could be altered with as little as 5° of pelvic rotation. The Bland–Altman plot identified only three outliers among the 51 hips included in our study. Therefore, we can assume that our correction of 0.9 for radiographic magnification and our setting of 4° for the perspective projection angle were appropriate. While errors are also possible on the CT image, using an analysis software can greatly correct these issues [31]. As there is a 3-mm limit between the slices, the image used for measurement may not exactly cross through the femoral head center. Additionally, the perspective projection angle of 4° was calculated only in Japanese patients, with the possibility of race-dependent differences. However, we observed a strong correlation between distances A’ and B. Therefore, it is reasonable to consider line α as the point of the ilioischial line at the CT slice level. The error from this method was within 2 mm, a level of accuracy sufficient for clinical applications such as preoperative planning of THA cup placement.

On the pelvic CT axial images, the cortical bone region at the tangent point of the medial acetabular wall and a line inclined at a 4° angle from a line perpendicular to the floor is the bony region of the ilioischial line. Therefore, the offset distance, based on the ilioischial line of the cup, can be measured on the CT axial slice. This method would provide accurate preoperative templating from radiographs and CT images without requiring specialized software, which may improve THA outcomes.

### Limitations

This study has some limitations. First, we did not evaluate the pelvic tilt on radiographs and CT images. Second, while it is desirable to calculate and measure the radiographic offset beam based on the size of each pelvis, this process is complicated in actual clinical practice. Therefore, this measurement was simplified to a value of 4°. However, in individual cases, the perspective projection angle ranged from 3.3° to 4.3°. This difference had little effect on distance B.

## Conclusions

The study showed that the ilioischial line is located where the line α intersects with the medial acetabular wall. Furthermore, in most cases, the cortical bone area representing the ilioischial line was present on the posterior half of the medial acetabular wall. This finding may provide useful information on the three-dimensional features of the acetabular structure in radiographs and CT images.

## Supplementary Information

**Additional file 1.**

## Data Availability

The data that support the findings of this study are available from the corresponding author, MK, upon reasonable request.

## Abbreviations

2D: Two-dimensional
AP: Anteroposterior
CT: Computed tomography
ICC: Inter-class correlation coefficient
MPR: Multiplanar reformation
SD: Standard deviation
THA: Total hip arthroplasty
